# Accelerated annealing of colloidal crystal monolayers by means of cyclically applied electric fields

**DOI:** 10.1038/s41598-021-90310-7

**Published:** 2021-05-26

**Authors:** Peng-Kai Kao, Bryan J. VanSaders, Sharon C. Glotzer, Michael J. Solomon

**Affiliations:** 1 Department of Chemical Engineering, University of Michigan, North Campus Research Complex, Building 10 – A151, 2800 Plymouth Road, Ann Arbor, MI 48109 USA; 2 Department of Materials Science and Engineering, University of Michigan, Ann Arbor, MI USA

**Keywords:** Colloids, Self-assembly

## Abstract

External fields are commonly applied to accelerate colloidal crystallization; however, accelerated self-assembly kinetics can negatively impact the quality of crystal structures. We show that cyclically applied electric fields can produce high quality colloidal crystals by annealing local disorder. We find that the optimal off-duration for maximum annealing is approximately one-half of the characteristic melting half lifetime of the crystalline phase. Local six-fold bond orientational order grows more rapidly than global scattering peaks, indicating that local restructuring leads global annealing. Molecular dynamics simulations of cyclically activated systems show that the ratio of optimal off-duration for maximum annealing and crystal melting time is insensitive to particle interaction details. This research provides a quantitative relationship describing how the cyclic application of fields produces high quality colloidal crystals by cycling at the fundamental time scale for local defect rearrangements; such understanding of dynamics and kinetics can be applied for reconfigurable colloidal assembly.

## Introduction

Because of their nano to micro dimensions, colloids crystallize more slowly than molecular, surfactant, or polymeric systems^1^. Electrokinetic^2–7^, magnetic^8^, and photo-induced^9^ methods have been used to drive colloidal self-assembly at accelerated rates. Yet these methods often confront a tradeoff between the rate of crystallization and the quality of the self-assembled crystal^10^. That is, colloidal crystals formed at faster rates are prone to include undesirable defect microstructures such as vacancies, dislocations, and grain boundaries, each of which has negative effects on the overall crystal quality^11^. Understanding how fields can be used to drive colloidal crystallization that is both fast and high quality can be applied to produce ordered arrays with collective properties of value to applications such as reversible structural color^12^, photonic polarization^13^, and shape-memory retention^14^.

In general, annealing refers to a variety of tactics used to improve the microstructure of a condensed phase by controlling the kinetics of diffusion. The most commonly encountered form of annealing is to heat a crystalline solid to a point where internal diffusion rates (and hence grain growth) are significantly accelerated, but below the point of thermodynamic melting. This procedure generally drives the microstructure of the material towards equilibrium, which is often desirable. Annealing has been widely used in metallurgy, macromolecular science, and biotechnology^15^. Steel, for example, is commonly thermally annealed to alter its physical and mechanical properties for commercial applications. In macromolecular crystallization, cryogenic flash-cooling can quickly form crystals for structure determination of biological macromolecules, yet it also dramatically increases the lattice misorientation (mosaicity) of the crystals^16^. The quality of flash-cooled crystals can often be improved by warming and then cooling for a short amount of time. This annealing procedure can drive local lattice reordering and reduce the distribution of lattice spacing and orientation.

In equilibrium systems, only thermal energy is available to drive transitions to resolve metastable defect states in favor of the free energy minimizing structure. Increasing the system temperature increases the rate of such transitions, as thermal energy is equipartitioned across all degrees of freedom. In out-of-equilibrium systems, however, energy can be introduced heterogeneously to specific degrees of freedom that may strongly drive the system towards new configurations^17–19^. Particles that are given just enough mobility to reconfigure their local neighborhoods (but not so much as to form a jammed state^20^ or to induce bulk melting) can accelerate the annealing of the colloidal crystals and create highly ordered crystals. Bevan and coworkers designed a closed-loop control scheme to evolve colloidal crystals from polycrystalline states to a single domain crystal under electric field mediated crystallization^21–23^. Active matter has also been applied to overcome naturally occurring kinetic barriers. For example, van der Meer showed via simulation, and Ramananarivo et al. by experiment, that the annealing of passive colloids can be accelerated by introducing self-propelled microparticles^17,24^. Singh et al. showed that the crystallization of passive silica colloids can be directed by a small number of active colloids^25^. Altemose et al. used light-powered oscillations of active matter to induce annealing of colloidal crystals^26^.

While accelerated annealing may be accomplished by injecting energy into the colloidal system via active particles or external fields, another alternative to introduce out-of-equilibrium fluctuations to the system is to cycle the potential interactions between colloids. This scheme is conceptually similar to temperature cycling for heat treatment annealing of metals^27^. In this context, strong field-induced colloidal interactions are equivalent to a low temperature state, and weak field-induced interactions to a high temperature state. Experimentally, Swan et al. showed that a pulsed uniform magnetic field can be used to escape kinetically arrested states and assemble paramagnetic colloids into crystalline domains^8^. Sherman and Swan showed via simulation of a colloidal crystal that a cyclically toggled external field leads to a faster growth rate and fewer defects formed when compared to self-assembly in a steady field^10,28^.

In this work, we present simulations and experiments to study the annealing of colloidal crystal monolayers by cyclic application of an AC electric field. We define the cyclically applied waveform by means of three parameters: amplitude, on-duration ($t_{on}$), and off-duration ($t_{off}$). Within this cyclic scheme, colloidal particles form close-packed structures during the $t_{on}$ period of the cycle; they are free to thermally diffuse during the $t_{off}$ portion of the cycle. We define the duty cycle as the ratio between $t_{on}$ and $t_{off}$, and vary this ratio as part of the study. We observe different annealing performance and rates for different duty cycles. Time-resolved confocal laser scanning microscopy (CLSM) and small-angle light scattering (SALS) measurements of the cyclic annealing demonstrate that local ordering precedes global ordering in the annealing process. In addition, we find that the best annealing performance, as measured by CLSM, occurs at a duty cycle time that is approximately one half of $\tau_{50}$ ($t_{off}$ = 0.5 $\tau_{50}$). Here $\tau_{50}$ is the half-life time of a crystal melting under the same field assembly conditions^29^. Using a molecular dynamics (MD) model of cyclically applied field-assisted crystallization, we observe a similar relationship between the best annealing performance duty cycle time and the characteristic system melting time. Furthermore, this duty cycle is robust to changes in the particle interactions. By tracking energy exchanged with the thermal reservoir during simulation, we observe a maximum in heat transfer (dissipated work) at slightly shorter duty cycle times than that of peak annealing. This difference is hypothesized to be due to diffusive motion on length scales less than a single lattice spacing. By contrast, defect annealing requires cooperative rearrangement over longer distances and times.

Our results suggest the existence of a fundamental relationship between the kinetics of melting and the characteristics of the cyclic field that optimize annealing. That is, colloidal crystal monolayers self-assemble rapidly and with high quality under cyclical loading at a duty cycle that coincides with the fundamental timescale for local defect rearrangements. At this timescale work dissipation rates are nearly maximized. This timescale-matching strategy represents a novel approach to annealing that could be easily extended to other types of systems at different length and time scales without the need to characterize or navigate the free energy landscape.

## Results and discussion

### Cyclic annealing of colloidal crystals with different duty cycles

**Figure 1:**
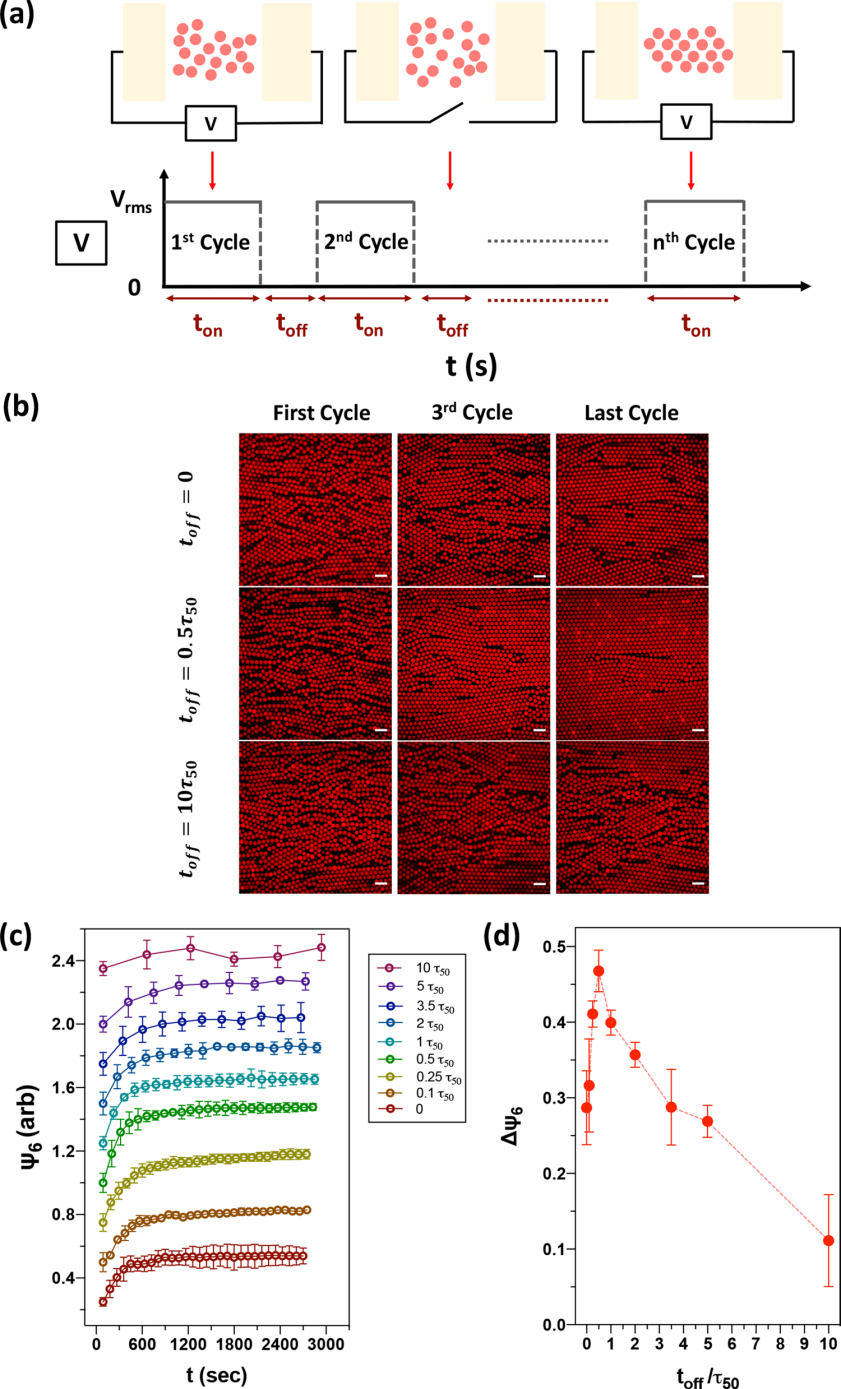
The impact of $$t_{off}$$ on colloidal crystal quality in cycled electric fields experiments. (**a**) Schematic illustration of the experimental procedure for cyclic annealing of monolayer colloidal crystals. The electric field is initially turned on and maintained at $$V_{rms}$$ = 8.0 V (*E* = 32 kVm^−1^) and  frequency 5 MHz for $$t_{on}$$; the electric field is then off for a period of $$t_{off}$$. The applied field is cycled for a duration of 2700 s. (**b**) 2D confocal laser scanning micrographs of $$t_{off} $$ = 0, 0.5 $$\tau_{50}$$ and 10 $$\tau_{50}$$ at the first, third and last cycle, respectively. (**c**) Time evolution of $$\psi_{6}$$ data for cycled fields experiments with nine different durations of off time. $$t_{on}$$ = 90 s for all experiments. Data in (**c**) are offset for clarity. (**d**) Change in $$\Delta \psi_{6}$$ as a function of melting time ratio $$t_{off} /\tau_{50}$$. The maximum $$\Delta \psi_{6}$$ occurs at $$t_{off} /\tau_{50} = 0.5.$$ Scale bars in CLSM images are 10 μm.

We study cyclic annealing through the crystallization of polystyrene spheres with diameter 4.00 ± 0.04 μm suspended in 0.1 mM NaCl aqueous solution. The colloidal suspension was injected into a coplanar AC electric field device to generate monolayer colloidal assemblies. Figure 1a illustrates the annealing experiment. During each cycle, the electric field is maintained at a constant root-mean-square voltage ($V_{rms}$) of 8.0 V, creating an electric field strength (*E*) of 32 kVm^−1^ and frequency 5 MHz for $t_{on}$, and then switched off for $t_{off}$. This field strength and frequency were selected to ensure that the colloidal particles assemble into dense 2D crystal structures^29^. Under these conditions the field-induced polarization of the colloids leads to particle chaining along the electric field direction, and eventual 2D crystallization along the bottom surface of the device.

We explored the impact of cycling the applied AC field on crystal annealing. During field-on conditions (of duration $$ t_{on}$$), polarization of the colloids induced particle attraction. Conversely, during field-off conditions ($$ t_{off}$$) particle dynamics were controlled by diffusion and hard-sphere like interactions. $$t_{on}$$ was set to be a constant value of 90 s for all experiments. This on-duration was selected as per previous work^29^, which indicated that the half-life of crystallization,$$ \tau_{crystal}$$, in this system is 14.8 ± 0.9 s. By applying the electric field for 90 s, we ensure that the system has completed its freezing transition during the on-phase of the cycle. During $$t_{off}$$, the electric field is turned off, and spheres relax and reconfigure under Brownian motion during this period. At the conditions of these experiments, the half-life for the melting transition ($$\tau_{50}$$) is 48.0 ± 1.7 s. The half-life times for crystallization and melting were measured based on the growth and decay of the light scattering diffraction response under identical external fields conditions by means of small-angle light scattering^29^. The duty cycle of the electric fields, $$\xi = t_{on} /\left( {t_{on} + t_{off} } \right)$$, is varied from 0.16 ($$t_{off}$$ = 10 $$\tau_{50}$$) to 1 ($$t_{off}$$ = 0) in this study. All experiments are performed for a total duration of 2,700 s.

Both $$t_{on}$$ and $$t_{off}$$ are independent parameters in the study. We observed the degree of annealing as a function of the cyclic duty cycle by specifically changing $$t_{off}$$ (with $$ t_{on} $$ fixed). We chose this design space so as to focus on the de-correlating effect of the $$t_{off}$$ period while ensuring that $$ t_{on}$$ is sufficiently long for a complete phase transformation to occur in the period of the cycle. The study can therefore be thought of as probing one region of a design space specified by $$t_{on}$$ and $$t_{off}$$. To analyze this process, we take CLSM images and SALS images at the end of each field-on period. Given the duration of the cycle, we analyzed a number of cycles varying from 6 to 30 over the range of conditions studied. Each condition was tested five times by CLSM, three times by SALS, and seven times by MD.

Figure 1b compares representative CLSM results at the end of each field-on duration, with $t_{off}$ = 0, 0.5 $\tau_{50}$, and 10 $\tau_{50}$. We show the first, third and last cycle of CLSM images to demonstrate particle structural arrangements that occur as time progresses. In the ‘always-on’ condition ($t_{off}$ = 0), a phase with high orientational order yet with kinetically arrested defects (vacancies and grain boundaries) was observed in the last cycle. When the system undergoes a continual cycle of crystallization with short periods of melting between each cycle, a close-packed monolayer crystal with minimal defects formed, such as shown in the case of $t_{off}$ = 0.5 $\tau_{50}$. For $t_{off}$ = 10 $\tau_{50}$, the system has sufficient time in the field-off condition to fully melt during each cycle, which allows new defects to be generated at each $t_{on}$. This condition yielded structures with poor crystal quality and abundant local vacancies and dislocations.

We calculate the area fraction covered by spheres as d_2D_ = N × S_a_/A, where N is the number of spheres in a CLSM image, S_a_ is the projected 2D area of a single sphere, and A is the area of a CLSM image. For $t_{off}$ = 0, the area fractions increase from 65 ± 1.9% to 73 ± 1.5% during the annealing process. For $t_{off}$ = 0.5 $\tau_{50}$, the 2D area coverage increased from 65 ± 2.5% to 81 ± 1.0%. For $t_{off}$ = 10 $\tau_{50}$, the area fraction only increases slightly throughout the process—from 66 ± 0.2% to 70 ± 1.7%. The increase of d_2D_ for all cases demonstrates that a more ordered system is created via annealing. The system is able to accommodate more particles within the same 2D area as a consequence of this ordering because the free space (area) is greater when particles occupy crystalline lattices than random configurations^30^. This statement aligns with our observation that the highest steady state d_2D_ value occurs at $t_{off}$ = 0.5 $\tau_{50}$ and the lowest d_2D_ value occurs at $t_{off}$ = 10 $\tau_{50}$. A comparison of how d_2D_ changes as time progresses for these three duty cycles is shown in Supplementary Fig. S1.

To quantify colloidal crystal quality, we compute the six-fold bond orientational order $\psi_{6}$ (see Materials and methods Section) for nine annealing experiments with different duty cycles. At the end of each $t_{on}$, we capture five CLSM images and calculate $\psi_{6}$ based on centroidal positions of the particles. The locations where these five images were taken are separated by at least 130 μm along the centerline direction to avoid duplicative characterization of the area. We compute the average value and standard deviation of $\psi_{6}$ for each cycle. The time-evolution of $\psi_{6}$ for each different duty cycle is shown in Fig. 1c. $\psi_{6}$ data sets are plotted from arbitrary starting positions for purposes of visualization. We observe that $\psi_{6}$ increases quickly and remains steady for spheres assembled under steady AC electric field ($t_{off}$ = 0). For $t_{off}$ = 0.5 $\tau_{50}$, $\psi_{6}$ improves rapidly and continues to increase gradually for the entire process. However, if the field is off for a long $t_{off}$ in each cycle, no apparent annealing effect is seen in $\psi_{6}$. For example, $\psi_{6}$ shows no detectable trend over a ~ 2700 s duration when $t_{off}$ = 10 $\tau_{50}$.

We further captured the time scale of the annealing process by regressing the time-resolved $\psi_{6}$ data with the following exponential plateau model:

1 $$ \psi_{6} = \psi_{6,M} - \left( {\psi_{6,M} - \psi_{6,o} } \right)e^{ - kt} = \psi_{6,M} - \Delta \psi_{6} e^{ - kt} $$

where $\psi_{6,M}$ is the maximum $\psi_{6}$, $\psi_{6,o}$ is the initial $\psi_{6}$, and $k$ is the annealing rate constant. We define $\left( {\psi_{6,M} - \psi_{6,o} } \right)$ as $\Delta \psi_{6}$, which is a convenient parameter to quantify the annealing performance.

Figure 1d demonstrates the significant dependence of $\Delta \psi_{6}$ on $t_{off} /\tau_{50}$, as extracted from Eq. (1). $\Delta \psi_{6}$ is singly peaked at $t_{off} /\tau_{50}$ = 0.5. The existence of a maximum in $\Delta \psi_{6}$ indicates that there is an optimal condition at which particles in the film are able to circumvent kinetically arrested states. At this special timescale, re-arrangements during the field-off condition do not hinder crystallization once the field is switched on, but rather enhance it. For $t_{off} /\tau_{50}$ values extending from 0.1 to about 2, the relaxation of the system from its initial condition to the fluid ground state (for the field off condition) was interrupted by turning the field back on. This partial melting followed by recrystallization represents a nonequilibrium kinetic pathway that is leveraged by cyclic annealing to enhance the quality of the assembled colloidal crystal, as quantified by $\Delta \psi_{6}$. However, for $t_{off} /\tau_{50}$ ≥ 5, new defects are increasingly created during each cycle’s crystallization, and therefore annealing is less successful than the optimal condition.

The slope of $\Delta \psi_{6}$ vs $t_{off} /\tau_{50}$ is asymmetric about its peak, with larger (smaller) changes in annealing rate observed for small (large) $t_{off}$. Because it is $t_{off}$ that is varied in Fig. 1d, the configurational changes leading to $\Delta \psi_{6}$ are driven by diffusion (as opposed to configurational changes driven by the external field). The magnitude of these changes should scale with the magnitude of particle displacement due to diffusion. For annealing to be successful, there must be a sufficient number of particle displacements of sufficient distance. Small diffusive rearrangements (as occur for short $t_{off}$) may not allow the configuration of the crystal to meaningfully change, and therefore defects are not annealed. Conversely, we expect that there is an upper threshold for particle displacements beyond which too much disorder is generated in the system, also resulting in unsuccessful annealing.

We measured the two-dimensional short-time self-diffusivity of the colloids, D_s_, to gauge the range of displacements that particles undergo during the field-off time. D_s_ can be determined from measurement of the mean-squared displacement (MSD), $<\Delta x^{2} \left( {\Delta t} \right)>$^31^. The MSD of all particles in the system during $t_{off}$ was computed from particle trajectories, as determined by *Trackpy*^32^. An average of 1,000 particle trajectories per sample were measured and a small correction for drift applied. D_s_ is related to the MSD through $<\Delta x^{2} \left( {\Delta t} \right)>= 4D_{s} (\Delta t)^{\alpha }$. In the case with best annealing performance, $t_{off}$ = 0.5 $\tau_{50}$, we find that $D_{s}$ = 0.013±0.002 μm^2^/s and α = 0.75 (Supplementary Fig. S2a). Here, the scaling exponent α is smaller than 1, indicating particles behave sub-diffusively, a typical occurrence in a crowded system^33^. From these measurements, the average MSD of a colloid by $t_{off}$ = 0.5 $\tau_{50}$ in the cycle is 0.67 ± 0.06 μm. This value is 17% of the diameter of a particle.

Particle trajectories can be further be used to compute the ensemble-averaged van Hove distribution of particle displacements. The displacement distribution for three different lag times, τ = 0.2 s, 1 s and 5 s is summarized in Supplementary Fig. S2b. The van Hove probability histogram data is fit with a Gaussian function^34^. We find good overall agreement between the histogram data and the fitting. Therefore, the diffusion process during the field-off time exhibits a normal distribution and the van Hove function can be expressed as^35^:

2 $$ G_{s} \left( {\Delta x,\Delta t} \right) = \sqrt {\frac{1}{{2\pi <\Delta x^{2} \left( {\Delta t} \right)>}}\exp \left[ {\frac{{ - \Delta x^{2} }}{{2<\Delta x^{2} \left( {\Delta t} \right)>}}} \right]} $$

We then compute the displacement distribution with field-off time equal to 0.1 $\tau_{50}$, 0.5 $\tau_{50}$ and 10 $\tau_{50}$ (Supplementary Fig. S2c). Specifically, for the best annealing performance (field-off time equal to 0.5 $\tau_{50}$) we find that half of the particles diffuse less than 0.36 μm (which is less than 10% of the particle diameter) and only 6% of particles diffuse to 1 μm (which is one-half of the particle radius). These results suggest that displacements on length scales less than a single lattice spacing are optimal for annealing. When larger percentages of particles can move over length scales approaching the lattice spacing during each $t_{off}$ period, annealing gains do not persist from cycle to cycle. Because the distance particles move on average is much smaller than the particle length scale, the coordinated motion of defects is a possible mechanism for the annealing effect. This scenario aligns well with a recent study of the dynamics of 2D crystals; in these cases, coordinated fluctuations of vacancies and interstitials are expected to play a critical role in melting^36^.

### Local defect rearrangement and kinetically arrested states

**Figure 2:**
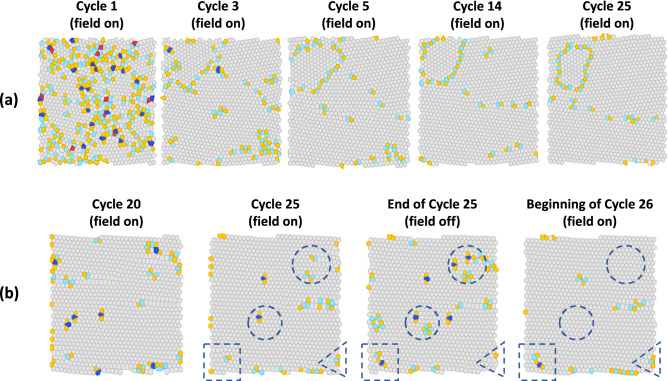
Voronoi analysis of colloidal crystals annealing. (**a**) Voronoi diagrams of experimental CLSM images under field-on condition with $t_{off} /\tau_{50} = 0.5$ at cycle 1, 4, 5, 14 and 25. (**b**) “On to off” and “off to on” Voronoi diagrams with $t_{off} /\tau_{50} = 0.25$ that show three types of defects rearrangement: recombination of defect pairs (circle), generation of new defects (square), and static defects (triangle).

To study the coordinated motion of defects, we identify local defect rearrangements and kinetically arrested states of colloidal particles during cyclic annealing by computing Voronoi diagrams for the CLSM images. Voronoi diagrams were computed using the library *Qhull*^37^. Particles within three diameters of the image boundary were not counted when tallying defects (these particles may lack neighbors due to the finite size of the image). Figure 2a shows the Voronoi diagrams of colloidal crystals that have been cyclically annealed at $t_{off} /\tau_{50} = 0.5$. These images were acquired at the end of each electric field-on period. Here, the gray cells represent particles with hexagonal neighbor shells while the red, yellow, light blue and dark blue cells represent four-, five-, seven- and eight-fold coordinated particles, respectively. The presence of topological defects—predominantly arrays of edge-sharing heptagon–pentagon dislocations—indicate the polycrystalline nature of the sample^38^. Many small crystal domains grow progressively between cycles and merge into larger single-crystal grains. By the conclusion of cycle 25, we observe two misoriented crystal domains separated by a low angle grain boundary ($\theta \sim13.4{}\pm0.8^\circ$). We also observe shrinkage of a closed grain boundary loop as time progresses (Fig. 2a, top left corner). We find that the total fraction of six-fold Voronoi cells in sample snapshots evolves in a similar manner to the $\psi_{6}$ bond order parameter (See Figure S3). Furthermore, the concentration of vacancies observed in experiment decreases initially but becomes constant at long times. This reflects that the cyclic annealing procedure accelerates the approach of the system to its thermodynamic ground state, which may include non-zero vacancy concentrations.

To further understand how topological defects are removed by annihilation, we study defect formation and motion during the switch from field-on to field-off (“on to off”) and vice versa (“off to on”). Figure 2b shows the transition Voronoi diagrams for colloidal crystals cyclically annealed at $t_{off} /\tau_{50}$ = 0.25. We captured three types of structural rearrangements at different sites: recombination of defect pairs (indicated by a circle), generation of new defects (indicated by a square) and conserved defects (indicated by a triangle). This example implies that recombination of dislocations generated by cyclic field switching is an important annealing mechanism. During the “on to off” switch, the system creates defects, then in the next “off to on” switch, those mobile defects (like dislocations) are driven by internal elastic forces to annihilate with preexisting immobile defects (like vacancies and grain boundaries). We observed that the motion of mobile defects can also be hampered by particle polydispersity, a nearby grain boundary, or multi-defect configurations that act as traps. As mobile defects are created, diffuse and annihilate with other defects, only a few kinetically arrested defects persist. Therefore, the efficacy of an annealing cycle depends on the creation of a limited number of mobile defects, which aid in annihilation, while avoiding the creation of additional immobile defects, which further reduce crystal quality.

### Molecular dynamics simulation of the cyclic annealing of a colloidal monolayer

In order to study the effect of variable cycling time on monolayer annealing, we also employed a MD model of the field-assisted assembly process. We follow a similar protocol as in previous studies^29^, in which particles under the influence of the driving field are simulated to represent induced polarization via discrete charges, with certain differences that are described in the Materials and methods section.

**Figure 3:**
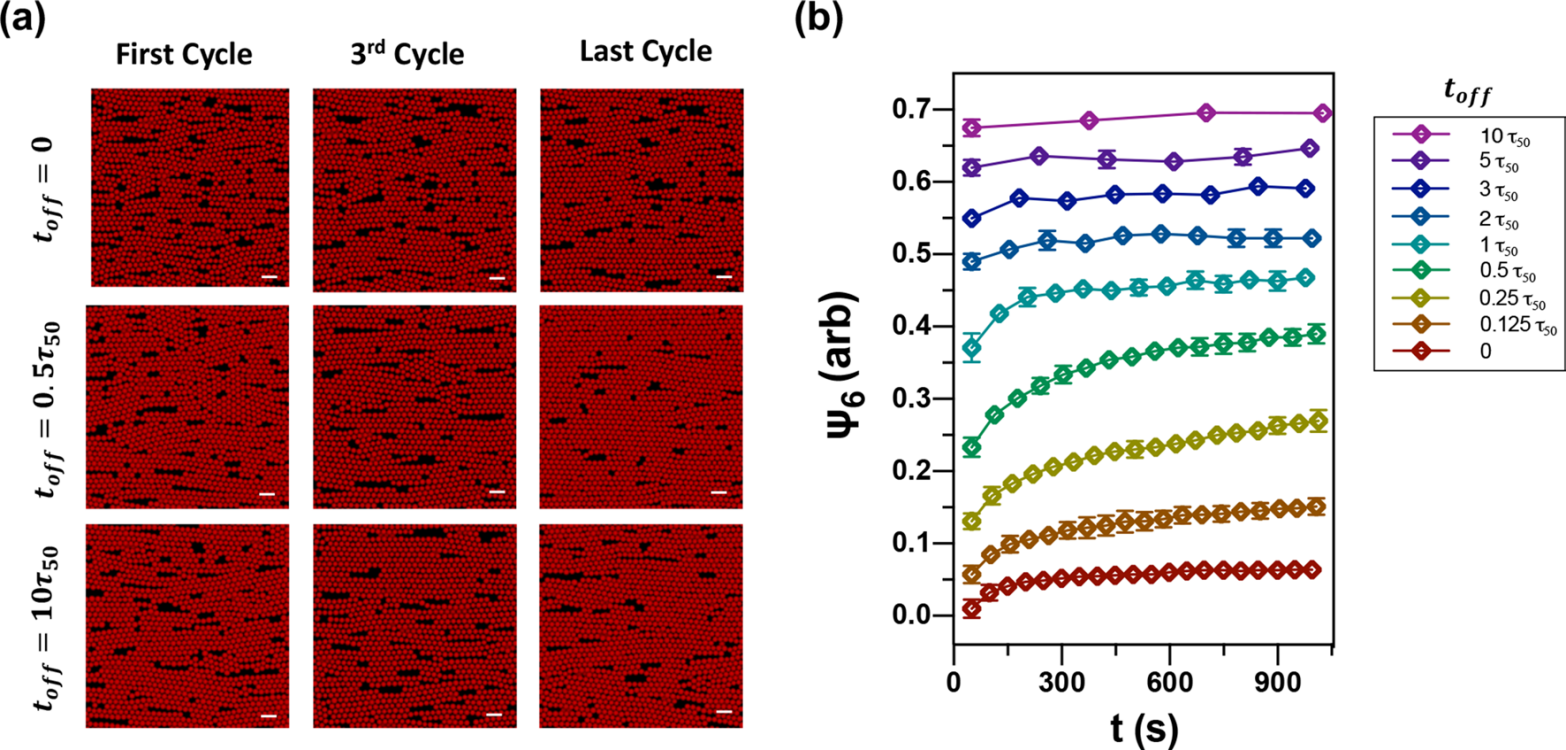
The impact of $t_{off}$ on colloidal crystal quality for simulated systems. (**a**) Images of simulated spheres assembled into dense crystals with $t_{off}$ = 0, 0.5 $\tau_{50}$ and 10 $\tau_{50}$ at the first, third and last cycle, respectively. (**b**) Time evolution of $\psi_{6}$ data for cycled-fields simulations with nine different durations of $t_{off}$. Data in (**b**) are offset for clarity. Scale bars in images are 10 μm. This data over the full range of long-cycle times can be found in Supplementary Fig. S4.

In Fig. 3a snapshots of simulated colloidal monolayers are shown for the first, third, and twentieth (last) cycles for different ratios of field-on to field-off. When expressed as fractions of the melting rate constant (calculated from a separate simulation), we find that a trend similar to experiments is observed. For constant field-on simulations, modest annealing of grain boundaries and vacancies occurs. For $0 < t_{off} < \approx \tau_{50}$ significantly accelerated annealing kinetics are observed. For $t_{off} \gg \tau_{50}$ , the film can nearly or completely finish its melting to a fluid state within the off period of a single cycle. In this limit, the crystallization of each field-on period is uncorrelated with previous cycles and only the thermal annealing that can occur during the field-on time is seen.

This observation can be quantified for simulated systems in the same manner as for experimental monolayers. Figure 3b shows the value of $\psi_{6}$ for a variety of $t_{off}$ conditions. Data points represent individual cycles, and error bars are the standard deviation of the mean as calculated from multiple locations within each simulated layer (see Supplementary Fig. S4 for additional data). As $t_{off}$ approaches 0.5 $\tau_{50}$, the improvement of $\psi_{6}$ after several cycles increases. For larger $t_{off}$ values however, the improvement in $\psi_{6}$ decreases, eventually approaching a flat curve with no improvement for $t_{off} = 10\tau_{50}$. This trend reproduces the behavior of the experimental films (Fig. 1c). Furthermore, we find that the optimal annealing off time ($t_{off} /\tau_{50}$ = 0.5) is insensitive to the microscopic details of the MD particle interaction model. Several models with different charged interaction strengths, isotropic particle attraction strengths, and electrophoretic force strengths were tested (Table S1), and the effect on system melting and freezing timescales is shown in Supplementary Fig. S5.

In our system, the dominant interaction driving self-assembly is the particle–particle dipolar interaction, consistent with reports in the literature for comparable systems^39–42^. As per the simulations, this dipolar interaction at contact is of scale 100 k_B_T. Applied AC electric fields such as used here have the advantage of permitting high electric field strengths while minimizing the effects of water electrolysis or electro-osmotic currents. There is some dielectrophoretic contribution to the self-assembly, as demonstrated by the fact that particle densities are highest at the midplane of the device; this contribution is included at a level that is about a factor of ten smaller than the dipole–dipole coupling. We emphasize that the general method of annealing advanced here is independent of the specific means by which the interaction strength is varied. Even in systems where attractions are not the result of induced polarizations, we propose that cycled annealing schemes can accelerate crystal quality.

Although the trend in $\psi_{6}$ is consistent between experiments and simulations, a notable difference between the two is the specific shape of the $\psi_{6}$ curves over the duration of the cyclic annealing. This can be seen by comparing the $t_{off} = 0$ curves from Figs. 1c and 3b. In the experimental case, the $\psi_{6}$ curves quickly reach a plateau and remain steady. In contrast, the simulated layers display an initial logistic rise in $\psi_{6}$ followed by a nearly linear increase at long times, especially for $t_{off} \sim0.25 - 0.5 \tau_{50}$. Thus Eq. (1) has a reduced quality of fit for simulated data in this $t_{off}$ range. This gradual rise indicates that annealing is still progressing at a measurable rate in the steady field-on condition for the simulations, unlike in the experiments. One possible interpretation of this trend is that low energy re-arrangements are occurring in simulations that are kinetically arrested or otherwise inaccessible in experiments.

How might low-energy arrangements proceed differently in simulation and experiment? We find that the strength of damping in the Langevin thermostat, as well as the energy scale of the interparticle attractions, are key variables controlling this long-time behavior. Langevin damping applies a drag force that is proportional to particle velocity. This parameter also controls, for instance, the terminal speed of particle settling in the simulation. For weaker damping and milder interparticle attraction, a greater rate of long-time annealing was seen. As our simulations lack frictional forces for particle–particle contacts, all dissipation occurs through the influence of the damping term. This velocity-based damping is always present, not only when particles are in contact. Frictional contact between particles would provide an additional barrier to the particle rearrangements needed for defect annealing and migration. Colloidal interparticle dissipation due to lubrication and viscous coupling has been measured^43^. This effect, along with direct particle–particle contact, due for example to surface roughness, have implications for the dynamics of dense suspensions^44,45^ and colloidal glasses^46^. Because Langevin damping is a global effect (and not confined to particle contacts) we were unable to fully explore the effect of particle–particle frictional interactions on annealing kinetics without also affecting other transport timescales in simulation.

An additional difference between the experiments and the simulations is that the latter lacks hydrodynamic interactions (HI) between the particles. HI is expected to not have a significant role on the equilibrium assembled structures but rather on the movements of the particles. HI complicates the relationship between structural and dynamic properties by having effects on both self- and collective diffusion of colloidal particles in suspensions^47^. For a 2D colloidal suspensions, Falck et al. reported the collective-diffusion coefficient is strongly coupled to HI^48^. It is possible that colloids in our experimental system undergo faster collective diffusion and reach kinetically arrested states faster. However, despite the difference in the origin of dissipation, the response of the systems to cycling is similar.

### Small angle light scattering

**Figure 4:**
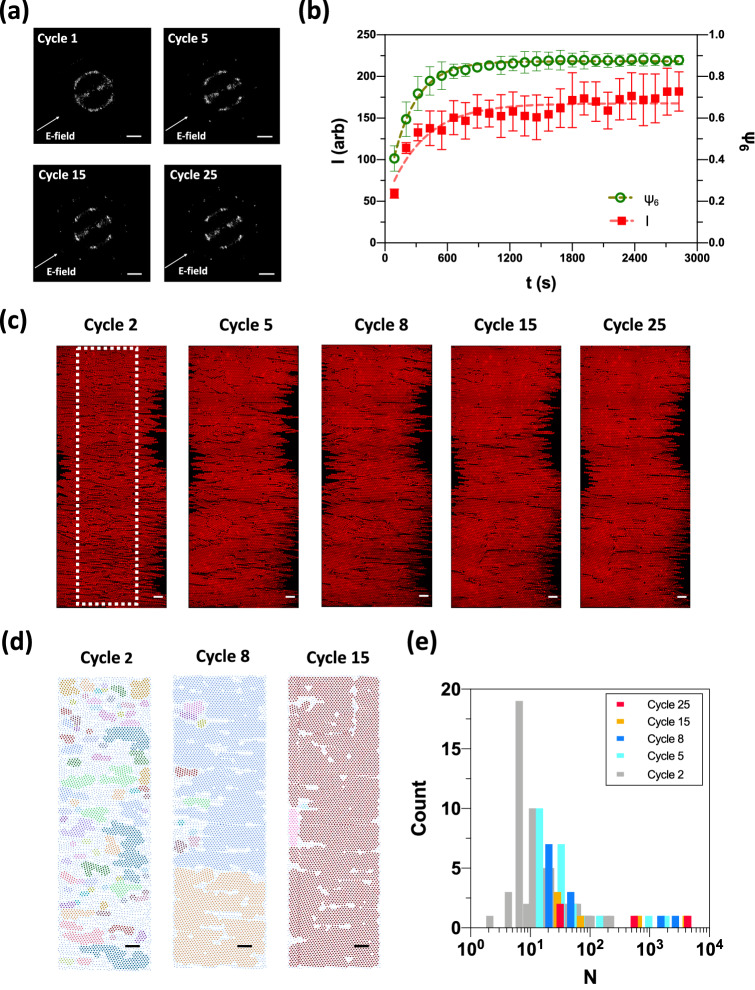
Local and global ordering characteristics of spheres under cyclic electric fields of $$t_{off} $$ = 0.5 $$\tau_{50}$$. (**a**) SALS images for cycle 1, 5, 15 and 25, respectively. (**b**) The change of peak intensity of light diffraction responses and the change of $$\psi_{6}$$ as a function of time t. The curves plotted are the mean and standard error of the mean for five and three CLSM and SALS experiments, respectively. (**c**) 2D confocal laser scanning micrographs for cycle 2, 5, 8, 15 and 25, respectively. The white dotted line encloses the centerline region for analyzing the distribution of crystalline grains. (**d**) Crystalline grains identified by particle proximity and local $$\psi_{6}$$ phase angle at the device centerline region for cycle 2, 8 and 15, respectively. (**e**) Histogram of the grain size (*N*) characterized for cycle 2, 5, 8, 15 and 25, respectively. Scale bars in SALS images are $$q = 1 \mu m^{ - 1}$$. Scale bars in (**c**) and (**d**) represent 20 μm.

We demonstrate the annealing of monolayer colloidal crystals and the kinetics of global annealing by SALS. Specifically, we measure the light scattering patterns at the optimal annealing condition, as per Fig. 1d. Figure 4a shows the time evolution of the SALS data. In the first cycle, we observe a six-fold symmetric light scattering pattern. The diffuse appearance of the light scattering pattern implies the formation of polycrystalline close-packed structure, each crystallite with a different orientation. In the fifth cycle, both the 1st and 2nd order scattering peaks can be clearly observed, indicating that a single crystal is developing on the scale of the scattering volume. The locations of these peaks represent the reciprocal lattice for a hexagonal close-packed (hcp) monolayer^49^. In the later cycles, such as cycle 15 and cycle 25, we are able to observe the 3rd order scattering peaks, which indicate that the single crystal is of increasingly high quality. We also observe a scattering pattern at low scattering wavevector (*q*, Materials and methods Section) oriented along the direction of the electric field. This scattering is a consequence of the device geometry. Upon the application of the electric field, long-range gradients in particle density are formed along the electric field direction between the electrodes, thereby introducing low *q* scattering features^29^.

The characteristic lattice spacing ($\sigma_{i}$) can be obtained from the SALS data based on the reciprocal relationship $q = 2\pi /\sigma_{i}$, where $\sigma_{i}$ represents the real-space distance. In Fig. 4a, $\sigma_{i}$ is 4.91 ± 0.04 μm at the first cycle and 4.92 ± 0.03 μm at the 5th cycle. As time progresses, $\sigma_{i}$ becomes 4.87 ± 0.07 μm at the 15th cycle and 4.86 ± 0.06 μm at the 25th cycle. The results show that the crystal quality improves without appreciable change in the average particle separation.

We analyzed the intensity of peaks in the first ring of the light scattering pattern to quantify crystalline order. Greater peak intensity represents higher quality global ordering of the monolayer crystal. Figure 4b compares the time-evolution of the SALS peak intensity $I$ and CLSM local order $\psi_{6}$ for $t_{off} /\tau_{50}$ = 0.5. During the annealing process, both peak intensity $I$ and local order $\psi_{6}$ increase monotonically as a function of time. By applying Eq. 1 to each data set, we determined $k_{SALS}$ and $k_{CLSM}$. The rate constant of local order annealing, $k_{CLSM, 0.5}$, is 0.0044 s^-1^ and the rate constant of global order annealing, $k_{SALS,0.5}$, is 0.0028 s^-1^, respectively. Our results show that short-range ordering achieves steady-state faster than long-range ordering. Therefore, local restructuring leads global annealing; the local rate is about 50% greater than the global rate.

To investigate the lag between local ordering and global ordering, an expanded real-space view of the annealed crystal was acquired by CLSM (image size of 250 μm × 600 μm). Figure 4c shows representative CLSM results during the field-on phase with $t_{off} /\tau_{50} = 0.5$. Although the size of these CLSM images is smaller than the beam size of the SALS device, they can be used to gain insight into the mechanism of crystallite growth that drives the global crystal quality characterized by CLSM. From cycle 2 to cycle 8, there are many small to medium sized crystallites separated by grain boundaries. After cycle 15, a significant reduction in the number of dislocation and grain boundaries was observed.

Six-fold coordinated particles are shown in Fig. 4d colored by grain membership. The identification and characterization of crystalline grains are described in the Materials and methods section. Many misoriented grains in cycle 2 merged into two main crystallites in cycle 8, where they gradually coarsen to one crystal after cycle 15. The early stage misoriented grains contribute to the spread in the light scattering diffraction peaks. Finally, we calculate the number of particles in each grain, *N*, and plot the distribution of *N* for each cycle in Fig. 4e. The histograms show similar distributions after cycle 15, indicative of the appearance of steady ordering in large grains. Based on the above analyses, we can conclude that the vast majority of particles formed local six-fold packings within misoriented grains in early cycles, followed by the merging and reorientation of those grains into large domain, perfect crystals. It is the lag in the annealing of the misoriented grains that generates the retarded kinetics of the (global) scattering relative to the (local) CLSM orientational ordering.

### Thermostat energy flow during cycled assembly

Over the course of cyclic annealing simulations, energy flow can be tracked into and out of the system. The Langevin thermostat that is used to maintain a constant temperature is also used to record energy flow between the system and a heat bath to which it is coupled. As the field condition is changed, kinetic energy must be added (in the case of field-on) or removed (field-off) in order to maintain a constant temperature. This energy flow indicates the total sum of work being done on the system by all sources. In this case those sources arise from the cycled field. We find that cycling conditions that lead to rapid annealing have the greatest rates of thermostat heat flow (dE/dt) into the system during the beginning of each cycle. For $t_{off} /\tau_{50}$ near 0.5, the drift in the configuration of the monolayer is such that a large quantity of work is done quickly to order the system—furthermore, the system is able to access lower energy configurations on each subsequent cycle. These observations indicate the specific nature of the coupling between the applied cyclic field, the dissipation by the monolayer, and the resulting configurational change as indicated by $\psi_{6}$.

**Figure 5:**
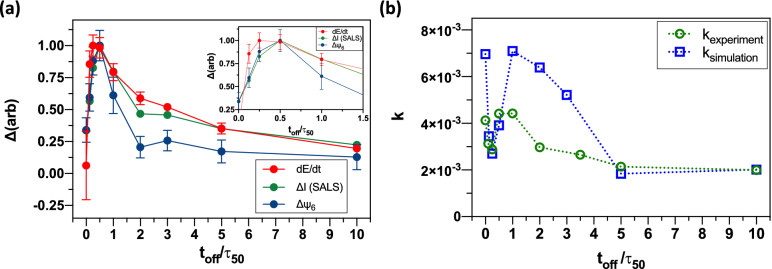
Performance and kinetics of colloidal crystals annealing as a function of melting time ratio $t_{off} /\tau_{50}$. (**a**) Relative changes of system order parameters as a function of $t_{off} /\tau_{50}$ for simulated systems. The difference of local order ($\psi_{6}$) and global order (*I*) are shown alongside rate of energy exchange with the Langevin thermostat. (*I* is measured by simulated SALS.) The change in $\psi_{6}$ is most sharply peaked around $t_{off} /\tau_{50}$ = 0.5. SALS peak intensity shows a somewhat broader peak distribution. Energy exchange rate with the thermostat is also broad; it furthermore shows a shift in peak position to $t_{off} /\tau_{50}$ = 0.25 (inset). (**b**) Relative changes of annealing rate constant *k* as a function of $t_{off} /\tau_{50}$ for both simulated systems and experiments. Here *k* has units of s^−1^.

For cycling times that couple weakly to the dynamics of the monolayer ($t_{off} /\tau_{50}$ < 0.25 or $t_{off} /\tau_{50}$ > 1.0), the rate of work (i.e., structural change to the configuration of the monolayer) is low. For $t_{off} /\tau_{50}$ < 0.25, only small changes to the configuration of the film have occurred during the field-off time, and so under field-on conditions only a small amount of work over a short time is done on the system to order it (Fig. 5a). Conversely, for $t_{off} /\tau_{50}$ > 1.0, the monolayer’s configuration has changed a great deal during the field-off time. When the field is switched on, the system slowly navigates a kinetically arrested energy landscape to return to the ordered state.

In Fig. 5a we plot the changes in order parameters for simulated systems as a function of $t_{off} /\tau_{50}$. The change in $\psi_{6}$ is most sharply peaked around $t_{off} /\tau_{50}$ = 0.5, and rapidly falls off to a low baseline at $t_{off} /\tau_{50}$ = 10. The SALS peak intensity follows a similar trend, while showing a broader peak distribution. We interpret this to be a result of the long-range (and therefore many-particle) nature of the SALS measurement. That is, for large $t_{off} /\tau_{50}$ local order around any given particle is not improved by cycling. However, long-range correlations, such as probed by SALS, are still impacted for such cycling. These long-range correlations (SALS) might be due to the correlations between the orientations of grains, which can develop because of the tendency of the field to favor alignment of the close-packed crystal directions. This effect is only observable at the scale of the entire system (i.e., via SALS). See Fig. 4c for examples of such grain alignment).

We also observe that the exchange of energy between the integrator and the particles in the simulated system is, unlike the other measures, peaked at $t_{off} /\tau_{50}$ = 0.25 (Fig. 5a). The energy exchange rate is the most local of the three measures, being dominated by nearest-neighbor interactions. That is, the heat flux from the system to the bath involves contributions from each of the two-body interactions between the particles. The local $\psi_{6}$, by comparison, requires the coordination of six neighbors around a particle. SALS further includes sensitivity to long-range correlations. From this data we see a general trend that short field-off times preferentially anneal pair-wise correlations in the monolayer, whereas larger field-off times affect more highly coordinated structures.

### Annealing kinetics

Previous computer simulations suggest that the operation of a cyclic field offers a simple and easily controlled scheme for creating colloidal crystals at a faster rate compared with steady-state assembly ^10^. In Fig. 5b we demonstrate the impact of $t_{off}$ on the annealing rate constant, acquired by fitting Eq. (1), for both experiments and simulation (see Supplementary Fig. S6 for examples of simulated data curves). For the experimental results, the maximum annealing rate appears at $t_{off} /\tau_{50}$ = 1. (Note in Fig. 5b that the larger the value of k the faster the dynamics of annealing.) A slightly lower annealing rate is observed at $t_{off} /\tau_{50}$ = 0.5, the condition that has the best annealing performance. A high rate constant also appears at $t_{off} /\tau_{50}$ = 0. Recall that this is the steady field condition. By comparison, as per the results of Fig. 1d, the crystal quality measure $\Delta \psi_{6}$ is comparatively low at $t_{off} /\tau_{50}$ = 0. These two results, taken together, indicate that spheres assembled under steady electric fields crystallize quickly; however, these structures are not high quality. The strong attractive forces that are induced by the field drive rapid crystallization, but do not promote reconfiguration and defect diffusion that supports further improvement in crystal quality. On the other hand, the simulations show that high quality crystals assembled at the optimal cyclic condition have lower k and therefore slower kinetics.

Figure 5b shows that similar trends in kinetics are present for both the experimental and the simulated systems, yet with quantitative differences observed near the optimal annealing condition. The magnitudes of the simulated rate constants are generally larger than those from experiment, with the exception of times near the optimal annealing cycling timescale ($t_{off} /\tau_{50}$ = 0.5). Near this region, the simulated annealing rate constants are reduced even more than the experimental ones. We interpret this result as follows: Due to the lack of frictional damping in simulations, they generally display faster particle dynamics. However, near the optimal annealing timescale, the simplified simulation model experiences a kinetic slowdown that is ameliorated by other factors in the experimental case. Although this remains a hypothesis, we propose that such a factor might be HI between particles.

The interpretation of the complex annealing kinetics behavior for experimental and simulated systems are as follows: The low annealing rates present near the optimal cycling timescale suggest that local configurational changes in the monolayer at these timescales are delaying the rise of $\psi_{6}$ without negatively impacting its eventual magnitude. Additionally, the trend of high rate constants at low $t_{off} /\tau_{50}$ is expected for the following reason: For short field-off times, the local particle configurations do not have enough time to significantly reconfigure. Therefore, when the field is turned on, the previous close-packed configurations are quickly recovered. Similarly, slow kinetics at large $t_{off} /\tau_{50}$ is also expected. At these conditions the local ordering of particles has sufficient time to relax to the fluid structure, and so when the field is turned on the close-packed configuration must be rebuilt from a completely disordered fluid state.

In conclusion, we have shown that colloidal monolayers assembled under AC electric fields exhibit significantly improved crystal quality under certain cyclic conditions. By using a local probe of structural order (CLSM) as well as a bulk measurement (SALS), we found that local ordering generally precedes global re-arrangement in this system. For both experiments and computer simulations we find that the cycling timescale that produces the highest quality crystals is similar to the fundamental characteristic timescale of crystal melting ($t_{off} /\tau_{50}$ = 0.5). By investigating the timescale of order parameter growth ($\Delta \psi_{6}$), the rearrangements and annihilation of mobile defects, and the heat exchange between the simulated system and thermostat, we find evidence that cycling timescales near $t_{off} /\tau_{50}$ = 0.5 activate coordinated reconfiguration mechanisms that progressively improve crystal quality over approximately 15 cycles. Our results suggest a general principle to design annealing by cycling induced potential interactions between particles. The cyclic conditions are optimized at a time that is approximately the characteristic melting time of the system. Future work could extend the annealing strategy developed in this report to encompass crystallite length scales greater than those investigated here (~ 250 μm), so as to address the dimensions needed for large-scale applications in fabrics, vehicles, and structures.

## Materials and methods

### Experimental design

A monolayer of colloidal polystyrene spheres (F8858, Invitrogen) was introduced in the coplanar AC electric field device, as shown in Supplementary Fig. S7. The device was prepared by deposition of Ti/Au electrodes onto a glass substrate, followed by cleaning the device in a freshly prepared base bath (1 N potassium hydroxide solution in isopropanol, Fisher Scientific) for thirty minutes before use. The electric field is cycled between $t_{on}$ and $t_{off}$ by means of an AC power source (RIGOL, DG1022). During $t_{on}$, a square wave with constant $V_{rms}$ 8.0 V and frequency 5 MHz was applied across the 250 μm gap between the electrodes, creating an electric field strength of 32 kVm^−1^. The electric field device is 1 mm in height, and it took one hour for particles to complete sedimentation. The initial number of colloids per unit surface area is 0.055 spheres μm^−2^, as determined by hemocytometry (NanoEnTek Inc.).

### Characterization of the colloidal assembly structure

CLSM is used (Nikon A Piezo z-drive, 100×, NA = 1.45 oil immersion objective) to visualize the particle-level microstructure. The image size is 512 × 512 square pixels, and the pixel size is 250 × 250 nm^2^. The centroid of any given particle in the images is identified with a resolution of ± 0.07 μm by means of the MATLAB circle detection function *imfindcircles*. To quantify the crystallinity, we calculated the six-fold bond orientational order $\psi_{6}$. For each spherical particle, $\psi_{6,j} = \frac{1}{{N_{j} }}\mathop \sum \limits_{k = 1}^{{N_{j} }} e^{{i6\theta_{jk} }}$ is computed based on N_j_ of nearest neighbors within the first peak of g(r), where θ_jk_ is the angle between a sphere j and its neighbor k with an arbitrary reference direction^50^. This analysis utilized the *freud* library^51^. We note that at this magnitude and frequency of applied electric field the colloids also undergo dielectrophoresis in addition to crystallization. The colloids are therefore more concentrated at the centerline between the two electrodes. In this study, we conducted microstructure characterization at the centerline region.

Colloidal particles are grouped into grains by clustering over a vector of their positions and $\psi_{6}$ phase angle (using the DBSCAN clustering algorithm^52^). We used a normalization where close-packed particles with no phase angle have a metric distance of 1. Clusters with a maximum member metric distance of 1.25 were computed. This value was found to produce good separation of grains; only nearest neighbors are considered as grain members (second nearest neighbor distance with no rotation under this metric is $\surd 3$), and orientational differences must be small. This procedure is an adaptation of Gray et al.^53^. Six-fold coordinated particles are colored by their grain membership.

### Analysis of light scattering response

SALS is used to quantify the global order of the colloidal crystal. The design of the SALS device is as in Kao et al.^29^. A laser (JDS Uniphase, 1135P) of wavelength 632.8 nm with a 1/e^2^ diameter of 0.71 mm was used as the light source. The analyzed radial width of the light scattering pattern was set to be 20 pixels to match the average width of the primary scattering patterns, which was 20 ± 3 pixels. This analyzed region corresponds to a scattering wavevector $q = 1.34 \mu m^{ - 1}$ in the radial direction and radial width $\Delta q = 0.17 \mu m^{ - 1}$. Here, the scattering wavevector, $q = \frac{4\pi n}{\lambda }{\text{sin}}\left( {\frac{\theta }{2}} \right)$, is equal to the difference between the incident wavevector and the scattered wavevector, where n is the effective refractive index of the sample and λ is the wavelength of the incident light. We then used locally weighted least squares smoothing (LOWESS) to fit the intensity data as a function of azimuthal angle to a Gaussian peak after baseline correction. The LOWESS peak intensity is used to quantify the light scattering response.

### Molecular dynamics simulation

MD simulation of cyclic, AC-electric field assisted self-assembly was conducted using HOOMD-Blue (v2.0)^54–56^. The interaction potential between particles is split into two components; a hard-sphere like repulsive force, represented by a Shifted Lennard–Jones potential^57^, and a screened dipole-like interaction represented by two discrete charge centers located within the interior of the repulsive particle core. The Shifted Lennard–Jones potential takes the form:

3 $$ V_{SLJ} \left( r \right) = 4 \epsilon \left[ {\left( {\frac{\sigma }{{\left( {r - \Delta } \right)}} } \right)^{12} - \left( {\frac{\sigma }{{\left( {r - \Delta } \right)}} } \right)^{6} } \right] $$

For $r < r_{cut} + {\Delta }$, and zero for larger distances. In this study $\sigma = 0.5$, and ${\Delta }$ was chosen so that the potential minima lay at $r = 2^{1/6}$ in simulation distance units. The potential was truncated at $r_{cut} = 2^{1/6}$, and shifted in energy so that $V_{SLJ} \left( {r = r_{cut} } \right) = 0$. For this interaction, *ε* = 1. These choices result in a potential that is purely repulsive and behaves more similarly to a hard sphere than the traditional Lennard–Jones potential with $\sigma = 1$^58^.

Particle interactions which modeled the effect of polarization were manipulated to represent the cyclically applied field. The discrete charge representation for the dipolar interaction follows the implementation of Crassous et al., which assumes that particle polarization due to the applied AC field is instantaneous and homogenous over the volume of the simulation^59^. Also included was an isotropic, short-ranged interparticle attraction, represented by a Shifted-Lennard Jones potential with $r_{cut} = 2.5*2^{1/6}$, and $\varepsilon = 2$. This isotropic attraction, as well as the anisotropic attractions due to screened dipole forces are the ‘polarization-induced’ interparticle forces used in this study.

The hard core and two charge-representing particles were simulated as a rigid body^60^, but without rotational freedom. This is because we assume the polarization direction of the particles to be fixed by the applied AC field direction. In the field-on condition, particles interacted via both hard-core repulsion and polarization-induced charge interactions. In the field-off condition, only the hard-core repulsion was simulated. All simulations employ Langevin integration at constant temperature. Particle masses are chosen to match 4 mm polystyrene particles. No hydrodynamic interactions between objects were considered explicitly in these simulations.

Particles were induced to settle onto a repulsive plane (with normal in the z-direction) by a constant force (with the strength of the gravitational force on 4 mm diameter polystyrene particles immersed in water at room temperature). In addition, in the field-on state a one-half wavelength sinusoidal potential of depth 8 $k_{b} T$ was applied across the simulation domain, parallel to the direction of particle polarization. This potential represents the effect of dielectrophoretic forces which drive particles towards the center of the device.

For each run 10,000 particles were simulated. To determine the crystallization and melting rate constants, simulations of duration equivalent to 150 s were performed and SALS curves fit according to the methods of Kao et al.^29^. Notably, we use here charge mediated interactions (of strength 100 $k_{b} T$) between particles that are significantly stronger than in reference^29^. This large interaction strength was necessitated by the large size of the particles used in this study. For 4 $\mu m$ diameter polystyrene particles immersed in water at room temperature, the energy of raising a particle by its own diameter against gravity is in excess of 10 $k_{b} T$. This interaction strengths brings the kinetics of the simulation into close agreement with observation (for simulations crystallization $\tau_{50}$ = 12.8 ± 1.2 s, compared to experimental $\tau_{50}$ = 14.8 ± 0.9 s). At these parameters, maintaining the field-on conditions for several $\tau_{50}$ yielded dense, polycrystalline monolayer films. Crystal grains within these films have characteristic sizes of approximately 10 particle diameters, similar to experiment. A system size of 10,000 particles therefore allowed several dozen to 100 grains to be observed simultaneously.

In the cyclic simulations, systems were held for 4.5 crystallization half-life times in the field on state, then in the off state for various fractions of the melting rate constant time. In addition to simulated SALS spectra, $\psi_{6}$ was calculated using the analysis package *freud*^51^*.* Similar to in the experimental case, several circular regions of radius 1200 mm near the dense center of the simulation domain were selected and averaged to obtain $\psi_{6}$. Lastly, the energy exchange with the Langevin thermostat was logged during numerical integration.

## Supplementary Information

Supplementary Information.

## Data Availability

All data generated or needed to evaluate the conclusions in the paper are available from the corresponding author upon request (mjsolo@umich.edu).
