# Supplementary material for: Accelerated annealing of colloidal crystal monolayers by means of cyclically applied electric fields
Source: Sci Rep. 2021 May 26;11:11042. doi: 10.1038/s41598-021-90310-7 (PMC8155009; doi:10.1038/s41598-021-90310-7)
Supplement: Supplementary file 1 — Supplementary Information. [file 41598_2021_90310_MOESM1_ESM.pdf]

Supplementary Information for:

Accelerated annealing of colloidal crystal monolayers by means of cyclically applied electric fields

Peng-Kai Kao,[1†] Bryan J. VanSaders,[2†] Sharon C. Glotzer,[1,2] Michael J. Solomon[1*]

[1]Department of Chemical Engineering, University of Michigan, Ann Arbor, Michigan.

[2]Department of Materials Science & Engineering, University of Michigan, Ann Arbor, Michigan.

[†]These authors contributed equally to this work.

[*]Corresponding Author:

Prof. Michael J. Solomon

Address: North Campus Research Complex, Building 10 – A151, 2800 Plymouth Road, Ann Arbor, MI 48109

Phone: 734-764-3119

Email: mjsolo@umich.edu

List of Supplemental Figures

Figure S1. Area fraction of assembled structures

Figure S2. Diffusion of particles during field-off time

Figure S3. Voronoi defect evolution

Figure S4. Local ordering in MD simulation

Figure S5. Annealing performance with MD model variations

Figure S6. Local and global ordering in MD simulation

Figure S7. AC electric field device

List of Supplemental Tables

Table S1. MD model parameter variations

Area fraction of assembled structures

The area fraction of spheres (d_{2D}) value represents how many colloidal particles assembled in the characterized region. A completer comparison of d_{2D} changes as time progresses with t_{off} = 0, $0.5\tau_{50}$, and $10\tau_{50}$ is reported.

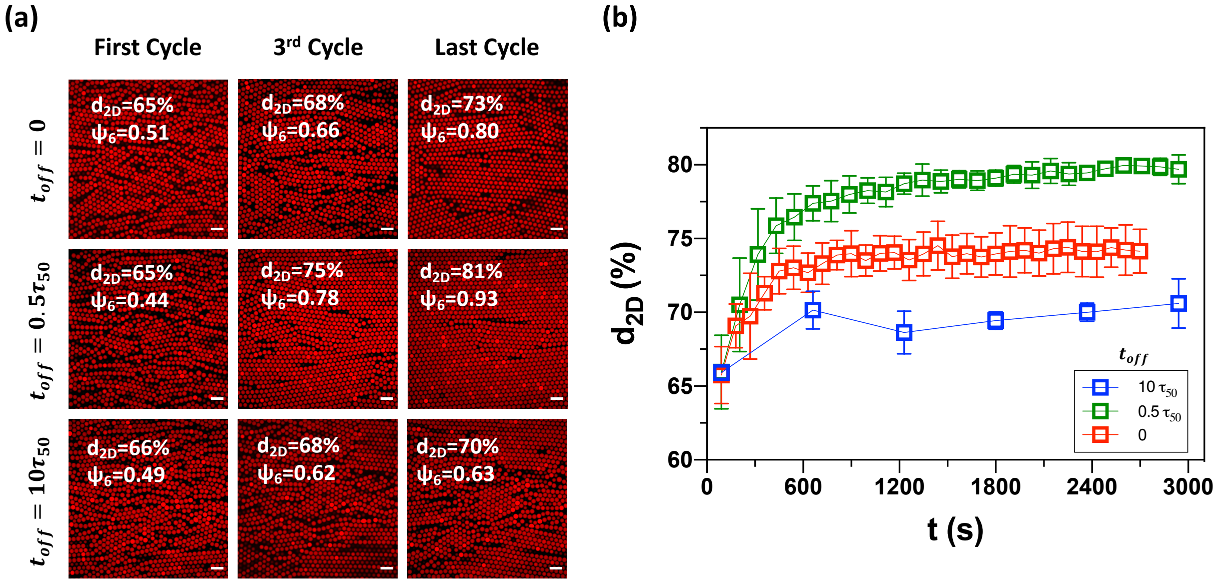

Figure S1. The change of area fraction of the colloids as time progresses. (**a**) Confocal laser scanning micrographs with calculated ψ_6 value and area fraction of spheres (d_{2D}) value for t_{off} = 0, $0.5\tau_{50}$, and $10\tau_{50}$ at the first, third, and last cycle, respectively. (**b**) Time evolution of d_{2D} for t_{off} = 0, $0.5\tau_{50}$, and $10\tau_{50}$. Scale bars in images are 10 µm.

Diffusion of particles during the field-off time

Under the best annealing performance condition $t_{off} = 0.5\tau_{50}$, there are about 6% of the particles diffused at a distance greater than 1μm during each field-off period (which is only half of the particle radius).

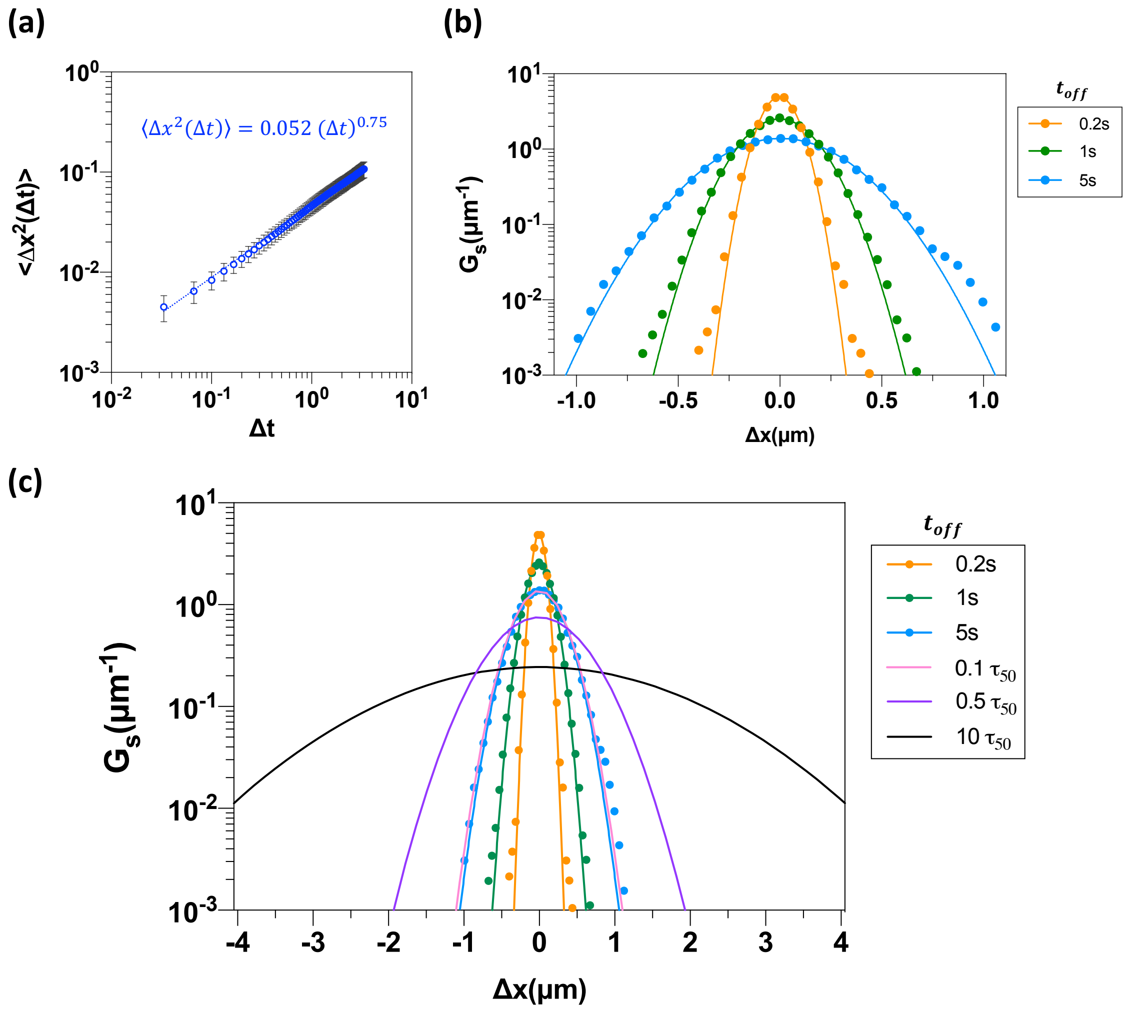

Figure S2. Two-dimensional short-time diffusion of particles under cyclic electric fields. (**a**) The MSD of particles in the first 10 s during field-off. (**b**) The van Hove distribution of particles in the first 0.2 s (orange), 1 s (green) and 5 s (blue) during field-off. (**c**) The computed displacement distribution based on Gaussian dynamics – as indicated per panel (**b**) – for field-off time equals to $0.1\tau_{50}$(pink), $0.5\tau_{50}$(purple) and $10\tau_{50}$ (black). Error bars are standard deviations from independent measurements.

Voronoi defect evolution

We track sample-wide changes to the number and area belonging to Voronoi defects for experiment and simulation. Defect area (A_{defect}) is defined as the summed area of all non-six-sided Voronoi cells. Additionally, the average area of all six-sided Voronoi cells is found (A_{ave}). The excess area belonging to defective particles is then approximated as: $A_e = A_{defect} - N_{defect}A_{ave}$. The quantity $\frac{A_e}{A_{ave}}$ then approximates the number of additional particles which could be added to the system if defective particles occupied the same area as non-defective particles. Expressed as a fraction of total snapshot particles, the quantity $f_{excess} = \frac{A_e}{A_{ave}N_{total}}$ is shown in Figure S3 (row a) for simulation (column i) and experiment (column ii). Simulations begin with a smaller fraction of excess area belonging to defective particles than experiments, and that area does not change much over the course of annealing. In contrast, experimental samples begin with a larger defect area and experience a reduction in defect area for annealing protocols which also improve the other measures (SALS, ψ_6) measured in this study. Together these results indicate that the proposed annealing strategy is effective at accelerating the approach of the system to a baseline defect concentration. The value of the baseline concentration is controlled by the thermodynamics of the crystalline state, and therefore we do not expect it to be modified by the annealing procedure. Figure S3 row b shows the fraction of six-sided Voronoi cells observed in snapshots during annealing. The fraction of six-fold Voronoi cells ($f_{6-sided}$) grows in a similar fashion to others measures of crystal quality.

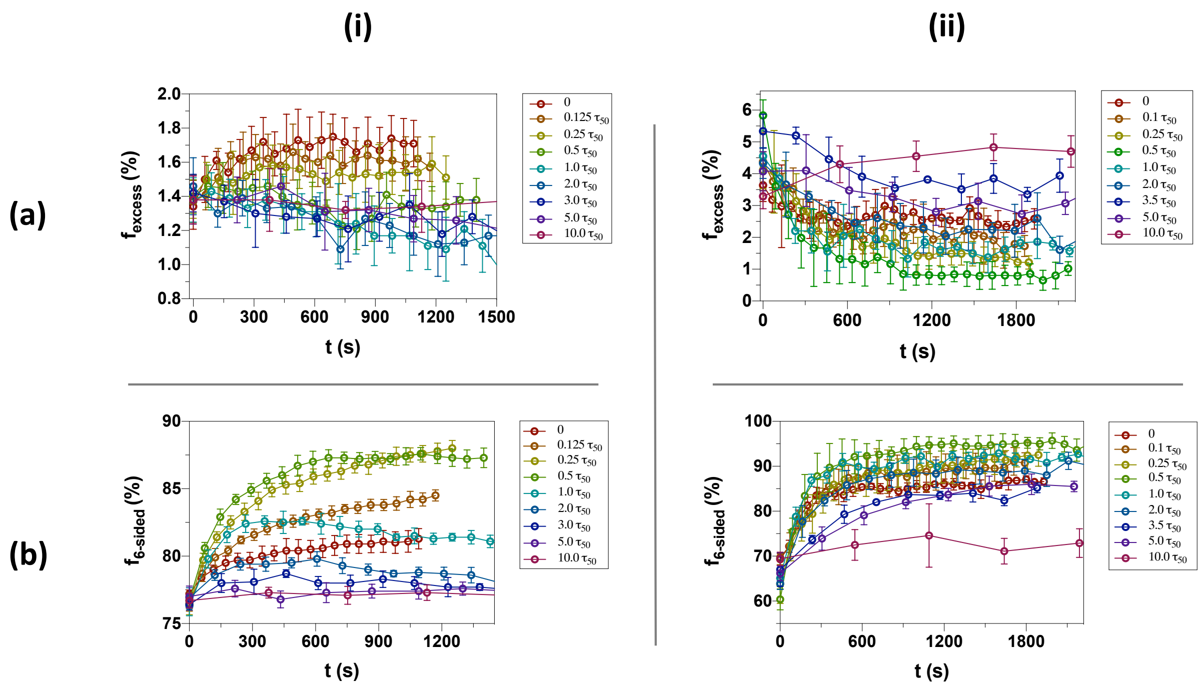

Figure S3. Evolution of defect and six-fold Voronoi cells during cyclic annealing. Row (**a**) excess area, which expressed as a fraction of particles that could be added if defective particle neighborhoods were transformed to ordered crystalline ones. Row (**b**) the fraction of six-fold Voronoi cells over the course of annealing. Column (**i**) reports simulation data; Column (**ii**) reports experimental data.

Local ordering in MD simulation

Time evolution of ψ_6 data for a simulated system showing twenty cycles of cyclic field annealing with nine different durations of t_{off}. For long t_{off}, little or no improvement in ψ_6 is seen over twenty cycles. As the t_{off} is decreased, the maximum improvement of ψ_6 is found for $t_{off} = 0.5\tau_{50}$. As the t_{off} is further decreased, the linear growth rate decreases and the ψ_6 improvement from zero to twenty cycles also decreases.

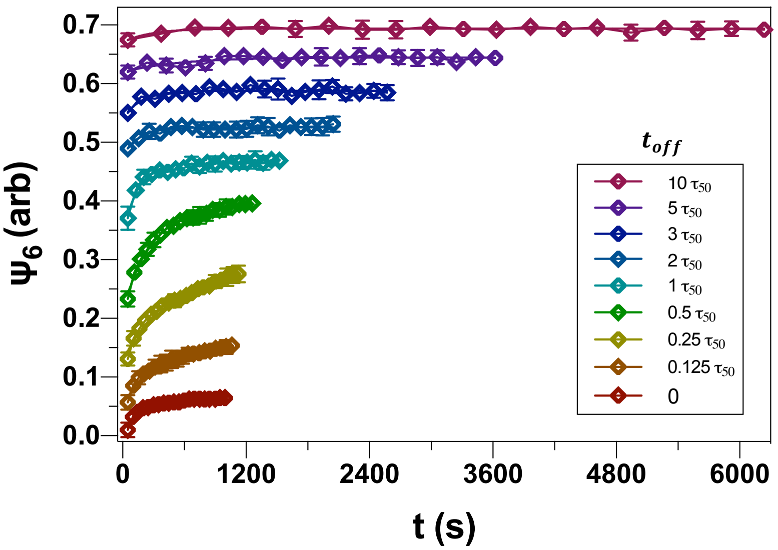

Figure S4. Local ordering over the full range of long-cycle times in MD simulation. Time evolution of ψ_6 data showing twenty cycles of cyclic field annealing. Curves are offset for comparison.

Annealing performance with MD model variations

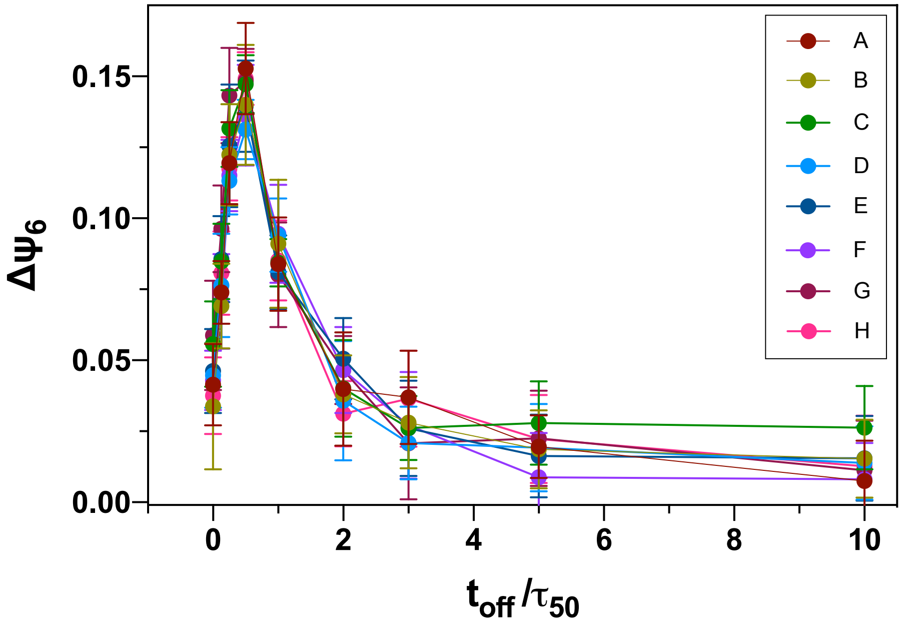

Figure S5. The impact of MD parameters on annealing performance in the simulated systems. Simulated $\Delta\psi_6$ changes over 20 cycles for MD models with a range of parameter values. The model parameters include electric field strength, interparticle attraction, and electrophoretic strength. Model parameter choice has some effect on peak shape, but for all models considered optimal annealing was found at $t_{off}/\tau_{50} = 0.5$.

Local and global ordering in MD simulation

Unlike in the experimental measurements, only negligible differences are present in the growth rate of SALS and ψ_6 curves in MD simulation. We attribute this difference to the comparatively smaller size of the simulated system, which is 250 µm x 500 µm as compared to 250 µm x 5000 µm for the experimental system. With fewer particles, the local and global re-arrangements are strongly coupled and have minimal differences in their rate of change.

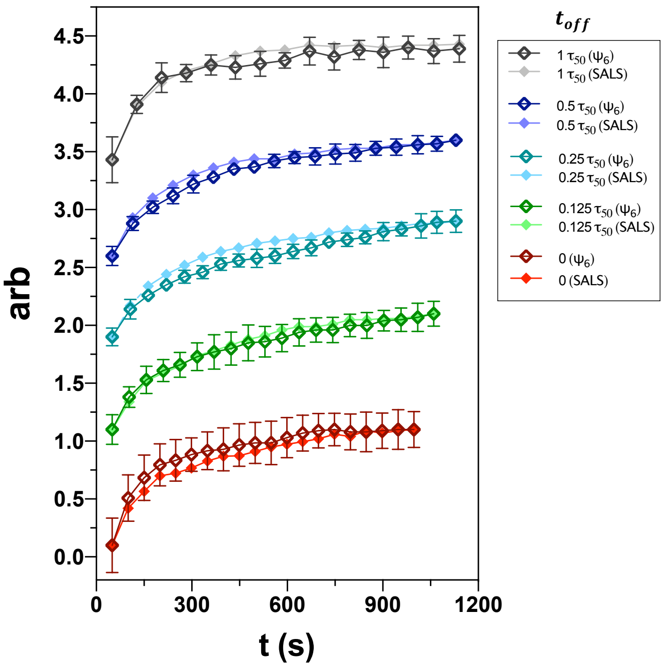

Figure S6. Comparison of local and global ordering in the simulated systems. Time evolution of ψ_6 data and SALS data for computer simulation system showing cyclic field annealing with five different durations of off-time. Curves are offset for comparison.

AC electric field device

The AC electric field device was prepared by first depositing 2.5 nm of titanium followed by 25 nm of gold onto a glass substrate. The gap between the two Ti/Au electrodes is 250 µm wide. The colloidal suspension was injected into the spacer, which is 1 mm in height. The inner diameter of the spacer is 5 mm.

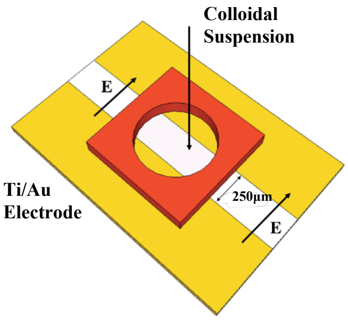

Figure S7. Illustration of the coplanar AC electric field device.

MD model parameter variations

Model	Electric field strength [x1/kT]	Attractive interparticle interactions [x1/kT]	Electrophoretic potential strength [x1/kT]	Crystallization half lifetime ($\tau_{crystal}$) [s]	Melting half lifetime (τ_{50}) [s]
O	100	2	8	12.8 +/- 1.2	31.9 +/- 3.1
A	150	1	8	10.3 +/- 1.3	27.6 +/- 3.6
B	200	1	8	9.0 +/- 1.7	24.5 +/- 1.8
C	150	1	16	10.5 +/- 1.25	27.8 +/- 2.7
D	200	1	16	10.4 +/- 2.25	24.5 +/- 2.4
E	150	2	8	11.4 +/- 1.7	28.5 +/- 3.6
F	200	2	8	8.6 +/- 1.1	23.7 +/- 2.6
G	150	2	16	10.5 +/- 1.0	29.5 +/- 2.5
H	200	2	16	10.2 +/- 1.9	24.8 +/- 3.1

Table S1. Model parameters for studying their impact on annealing performance in the simulated systems. Additional MD simulation models by variation of potential parameters controlling the electric field strength, interparticle attraction, and electrophoretic strength. The model used in the body of the text is model O. Half lifetimes here are reported in simulation units. The screening length for particle interactions is two particle diameters in all cases.
